# Effect of the Y_2_O_3_ Promoter on the Structure of the Ni/Al_2_O_3_ Catalyst for Ethanol Autothermal Reforming

**DOI:** 10.1021/acsomega.5c08809

**Published:** 2025-12-11

**Authors:** Augusto P. Cambunda, Maíra O. Palm, Diego A. Duarte, Rafael C. Catapan, Bruno F. Oechsler

**Affiliations:** † Graduate Program in Chemical Engineering (POSENQ), Federal University of Santa Catarina, Florianópolis, Santa Catarina 88040-900, Brazil; ‡ Graduate Program in Mechanical Science and Engineering (POSECM), Federal University of Santa Catarina, Joinville, Santa Catarina 89219-600, Brazil; § Physics Graduate Program (PPGF), Santa Catarina State University of, Joinville, Santa Catarina 89219-710, Brazil; ∥ Graduate Program in Mechanical Engineering (POSMEC), Federal University of Santa Catarina, Florianópolis, Santa Catarina 88040-900, Brazil

## Abstract

The Ni/Al_2_O_3_ catalyst is a viable alternative in catalytic reforming due to its excellent ethanol conversion and lower cost; however, it still suffers from deactivation by coke deposition. Doping the Ni/Al_2_O_3_ catalyst with yttrium oxide (Y_2_O_3_) neutralizes the acidic sites of the support, mitigating the reactions that lead to deactivation. In this work, Ni/Al_2_O_3_ catalysts with 1% and 2% Y_2_O_3_ were prepared by the wet impregnation method, dried at 90 °C for 24 h, and calcined at 975 °C for 5 h. The catalysts were characterized by N_2_ physisorption (BET), X-ray diffraction (XRD), scanning electron microscopy (SEM), energy-dispersive X-ray spectroscopy (EDS), temperature-programmed reduction (TPR), and H_2_ pulse chemisorption. The addition of the promoter reduced the temperature required for the reduction of nickel oxide (NiO) to Ni^0^ due to the lower formation of NiAl_2_O_4_ spinel. The Ni(10%)/Y_2_O_3_ (1%)—Al_2_O_3_ (S3) catalyst achieved 100% ethanol conversion and superior selectivities for H_2_ and CO_2_ during 480 min at 600 °C, with an H_2_O/C_2_H_5_OH/O_2_ molar ratio of 3:1:0.5 and a space velocity of 1300 h^–1^. Thermogravimetric analysis (TGA) indicated low coke formation (<1 mg_coke_/mg_catalyst_) for all catalysts and showed that the catalyst doped with 1% Y_2_O_3_ did not exhibit mass gain associated with the oxidation of Ni^0^ to NiO. Therefore, Y_2_O_3_ proved to be an effective promoter for increasing the activity and stability of Ni/Al_2_O_3_ catalysts in the autothermal reforming of ethanol.

## Graphical abstract

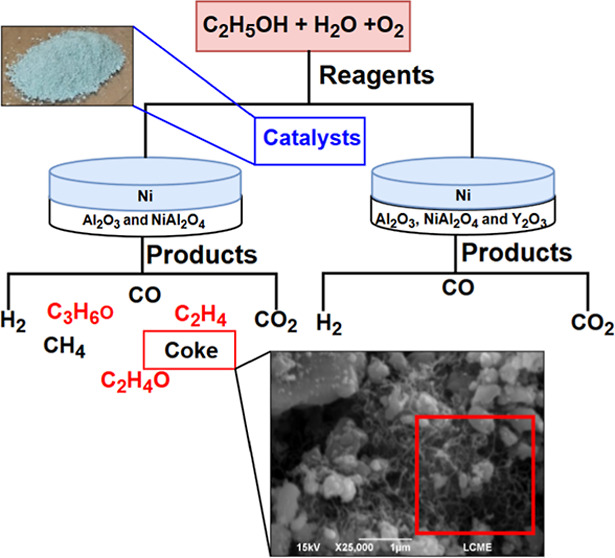

## 1 Introduction

The conversion of ethanol into hydrogen through autothermal reforming represents a promising alternative for clean energy generation in solid oxide fuel cells (SOFCs).^1,2^ This reaction occurs at moderately high temperatures, allowing the thermochemical utilization of waste heat from exhaust gases for the production of steam and ethanol vapor in generators and evaporators, increasing the overall efficiency of the process.^3–6^ The resulting fuel is rich in hydrogen, which can be directed to SOFCs as well as burners or combustion systems, promoting efficient energy recovery and contributing to the reduction of carbon emissions in automotive and distributed generation applications.^4^

Autothermal reforming has received great attention over the last years since it is considered a viable process for generating H_2_ for fuel cell system.^7–10^ The advantage of the process is related to its thermally neutral behavior (eq 1). In reforming reactions over heterogeneous catalysts, the mitigation of coke formation on the metal surface is one of the main challenges to keep the catalyst stable and active during the operation.^4,11,12^

1 $$C_{2}H_{5}OH_{\left(g\right)}+{0.5O}_{2\left(g\right)}+{2H}_{2}O_{\left(g\right)}↔{2CO}_{2\left(g\right)}+{5H}_{2\left(g\right)},\Delta H_{r}^{0}=-68\;kJ\;{mol}^{-1}$$

Nickel-based catalyst is widely used in autothermal reforming reactions, as it exhibits excellent catalytic performance. On the other hand, it has the disadvantage of fast deactivation by carbon deposition.^13–16^ In addition to Ni-based catalysts, noble metals are also widely employed.^17–19^ Noble metal catalysts have good catalytic activity and reduce the production of unwanted products, such as ethylene, a coke precursor that causes catalyst deactivation. However, the high cost of these metals makes them disadvantageous for industrial-scale applications.^9,20–22^

The use of promoters, including alkaline and rare earth elements (Y_3_O_2_, CeO_2_, La_2_O_3_), have been introduced to improve the stability of nickel-based catalysts.^13^ Doping Ni-based catalysts with yttrium oxide (Y_2_O_3_) increases the resistance by carbon deposition on the catalyst surface, the reducibility of nickel oxide (NiO), and in turn improves the catalytic performance.^23–25^ According to Li and Gong,^26^ the addition of rare earth elements as promoters to catalysts increases metal dispersion in reforming processes. The use of Y_2_O_3_ as a promoter is effective for the dehydrogenation reaction compared to other oxides formed by the elements of Lanthanide.^23^

The main challenges involve maximizing hydrogen yield and minimizing the formation of undesirable products, such as coke precursors, which lead to catalyst deactivation. This problem can be mitigated by developing appropriate low-cost catalysts. NiAl_2_O_4_ spinel, formed at high calcination temperatures, has been widely used in reforming reactions due to its resistance to sintering, coke formation, and thermal stability.^27^ The formation of this phase reduces the acidity of the Ni/Al_2_O_3_ catalyst, resulting from the lower amount of free Al_2_O_3_, which reduces the rate of ethylene formation and, consequently, carbon generation.^28^ Furthermore, after the reduction of NiAl_2_O_4_, the resulting metallic nickel presents smaller and more uniform crystals, with a more homogeneous distribution over the Al_2_O_3_ support, compared to catalysts prepared by impregnation with similar nickel content. This characteristic is advantageous for obtaining higher H_2_ yields.^27–30^

The catalysts’ performance are directly related to the implemented reaction conditions and the chosen catalyst. Ethanol reforming reactions at low temperatures favor the formation of carbon from the Boudouard and reverse carbon gasification reactions, while the hydrocarbon decomposition reactions occur at higher temperatures.^31,32^ However, to mitigate the reactions that favor the formation of carbon on the catalyst surface, the operation must be carried out within the oxygen/ethanol ratio range between 0.1 and 0.7, steam/ethanol ratio greater than 1.5, temperatures between 200 and 800 °C, and using a support such as Y_2_O_3_ that has a high oxygen storage capacity, due to its ability to reversibly change its oxidation states through oxygen storage/release. This property allows oxygen to react with carbon deposits, inhibiting the deactivation of reforming catalysts.^24,33–35^

Despite the high acquisition cost, the use of Y_2_O_3_ as a promoter in reforming catalysts can be economically advantageous in long-term applications.^36,37^ Its high stability and regeneration capacity reduce the need for replacement and operational downtime, resulting in lower overall process costs.^38^ Thus, the initial investment is offset by the greater durability and efficiency of the catalyst, making the use of Y_2_O_3_ a viable strategy for economic optimization on an industrial scale.^39,40^

In this context, we investigated the influence of the NiAl_2_O_4_ spinel, formed during high-temperature calcination = 975 °C, on the structure of the alumina-supported nickel catalyst (Ni/Al_2_O_3_). Furthermore, we evaluated the effect of the addition of yttria oxide (Y_2_O_3_) on the stability of the Ni/Al_2_O_3_ catalyst, particularly regarding its resistance to deactivation by coke formation during the autothermal ethanol reforming (ATR) reaction. The influence of Y_2_O_3_ on the formation of the NiAl_2_O_4_ spinel, especially during the calcination step at temperatures above 900 °C, was explored in detail. Studies such as this are essential in heterogeneous catalysis, as they allow a comprehensive understanding of the role of promoters in the structure and performance of catalysts. This work aims to evaluate the catalytic activity, focusing on the effect of the amount of Y_2_O_3_ on ethanol conversion, H_2_ production, ethanol dehydrogenation and dehydration reactions, and coke formation. The article covers the preparation of the catalysts, their characterizations, and the tests of catalytic reactions.

## 2 Experimental Section

### 2.1 Preparation of the Al_2_O_3_ Support

The Al_2_O_3_ (mixed oxide) support was obtained by precipitation from a solution of aluminum nitrate nonahydrate (Al­(NO_3_)_3_·9H_2_O). The solution was homogenized and maintained at 60 °C, under magnetic stirring on a hot plate, until complete evaporation of the water. The resulting solid was then dried at 90 °C for 24 h and subsequently calcined at 975 °C. It should be noted that the support was prepared without the addition of other compounds, such as Y­(NO_3_)_3_·6H_2_O or Ni­(NO_3_)_2_·6H_2_O, with the aim of exclusively evaluating the textural properties of pure Al_2_O_3_.

### 2.2 Preparation of Catalysts

Table 1 presents the catalysts prepared by wet impregnation using a metallic solution of nickel­(II), nitrate hexahydrate (Ni­(NO_3_)_2_·6H_2_O) (supplied by Exodus Scientific, sulfate content equal to 0.001%). The catalysts were supported on aluminum oxides, i.e., α-Al_2_O_3_ and γ-Al_2_O_3_ supports were precipitated from a solution of aluminum nitrate nonahydrate (Al (NO_3_)_3_·9H_2_O). The synthesis procedure consisted of the addition of (1 mol L^–1^) of Ni­(NO_3_)_2_·6H_2_O into Al­(NO_3_)_3_·9H_2_O (precursor of α-Al_2_O_3_ and γ-Al_2_O_3_) or α-Al_2_O_3_ powder (supplied by Almatis, with particle diameters ranging from 0.5 to 2.5 μm). The solution and suspension were then homogenized and maintained at 60 °C on a hot plate with magnetic stirring until the water was completely evaporated. Subsequently, the impregnated supports were dried at 90 °C for 24 h in a natural convection oven. The impregnated and dried materials were calcined in a furnace at 975 °C for 5 h with a ramp of 10 °C min^–1^ in a static atmosphere.^41^ The catalyst supported on α-Al_2_O_3_ (Ni 10%/α-Al_2_O_3_) was named as S1, while the catalyst supported on the precursor Al­(NO_3_)_3_·9H_2_O (Ni(10%)/Al_2_O_3_) was named as S2.

**Table 1:** Nomenclature and Nominal Composition of Synthesized Catalysts

catalyst	Ni mass content (wt %) (active phase)	support	Y_2_O_3_ mass content (wt %) (promoter)
S1	10	α-Al_2_O_3_	
S2	10	Al_2_O_3_	
S3	10	Al_2_O_3_	1
S4	10	Al_2_O_3_	2

Amounts of 0.0679 and 0.1357 g of Y­(NO_3_)_3_·6H_2_O were coprecipitated in two beakers containing solutions of Al­(NO_3_)_3_·6H_2_O. The resulting solutions were homogenized and maintained at 60 °C under magnetic stirring on a heating plate until complete evaporation of the water. The materials obtained after homogenization were dried at 90 °C for 24 h and subsequently calcined at 975 °C. The resulting supports, consisting of mixed oxides of Al_2_O_3_ and Y_2_O_3_ (with 1% and 2% by mass of yttria, respectively), were then impregnated with a metallic solution of Ni­(NO_3_)_3_·6H_2_O, according to the methodology described by Garbarino et al. (2015).^41^ The catalysts containing 10% by mass of nickel and different yttria contents (1% and 2%) were named S3 and S4, respectively. Figure 1 shows a representative scheme of the catalyst synthesis process.

**Figure 1:**
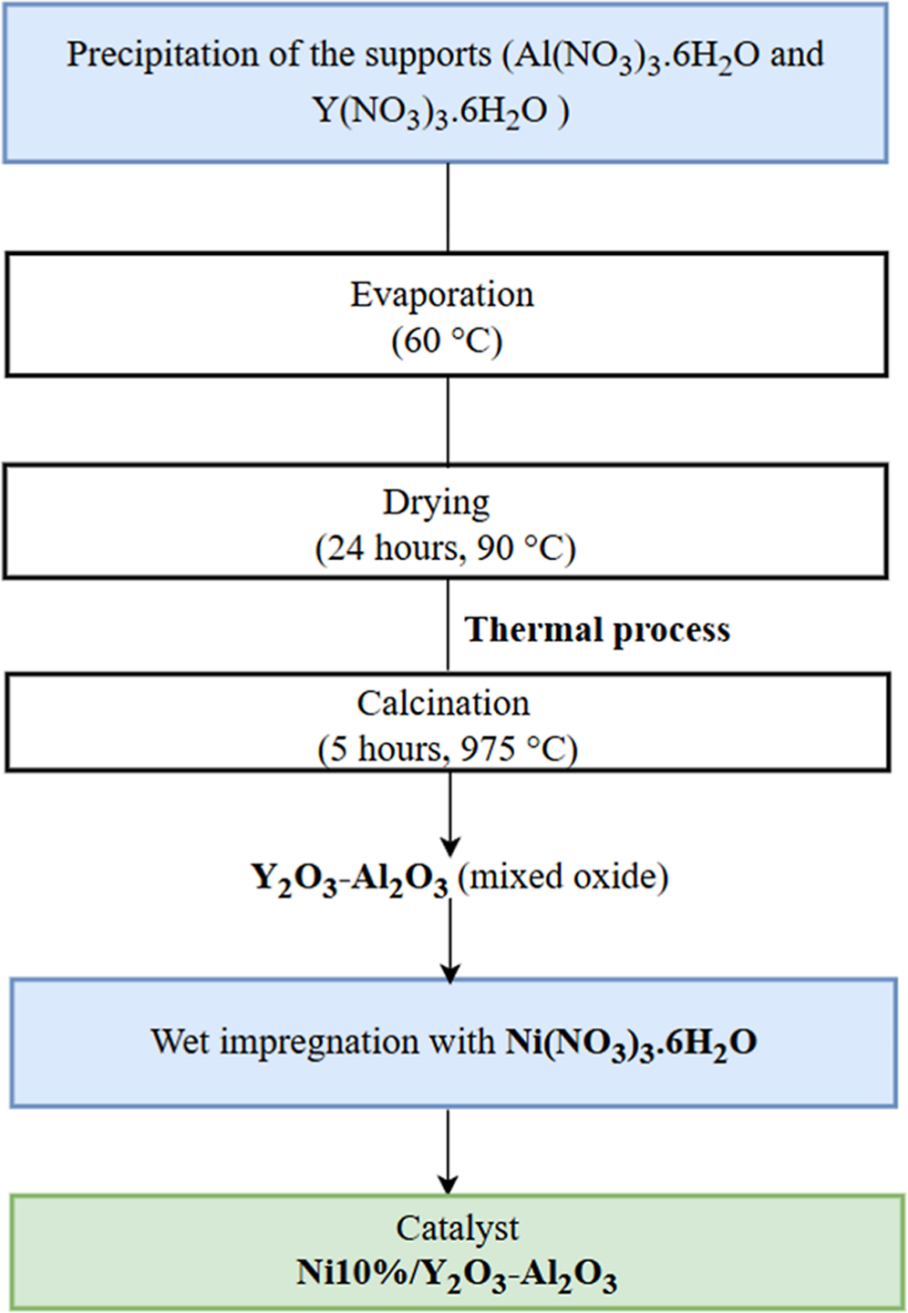
Schematic representation of the catalyst synthesis process.

### 2.3 Catalyst Characterization

The textural properties of the catalysts, including specific surface area (BET), specific volume, and average pore diameter, were obtained using the physical nitrogen adsorption technique (Autosorb-1 equipment—Nova 1200*e*, Quantachrome Instruments, USA). Initially, the catalyst sample (approximately 0.244 g) was subjected to vacuum pretreatment under N_2_ (99.9% purity) flow (300 °C for 24 h), to remove moisture and any volatile contaminants adsorbed in the sample. After the impurity removal step, the adsorption and desorption isotherms in liquid nitrogen at 77 K (−196 °C) were measured under different relative partial pressure conditions (*P*/*P*_0_ between 0 and 0.99). The X-ray diffraction (XRD) technique was used to study the crystalline structure of the synthesized catalysts. It was carried out on a SHIMADZU model XRD-6000 diffractometer, Cu Kα radiation (40 kV and 30 mA), scanning speed of 2.0 deg/min, and data collected in the range of 10.00° ≤ 2θ ≤ 80.00° (sampling pitch of 0.02°). Rietveld refinement was performed using X’Pert HighScore Plus software, and the results were compared to diffractograms from the PDF-2 database of the International Centre for Diffraction Data (ICDD), as presented in the Supporting Information. The average crystallite size was calculated from the Scherrer eq (eq 2)­

2 $$L=\frac{\kappa \lambda }{\beta \;\mathrm{Cos}\;\theta }$$

where *L* is the crystallite size, κ is Scherrer’s constant and assumes the value 0.89, λ is the X-ray wavelength (1.5406 Å for Cu Kα) and β is the full width of the peak corresponding to half the peak height.

The morphology and composition of the studied catalysts were obtained by field emission scanning electron microscopy and energy dispersive spectroscopy (FESEM/EDS, JSM-6390LV JEOL, Japan). Images with 10,000- and 15,000-times magnification were obtained with 15 kV accelerating voltage, and the samples were coated with gold. The reduction profiles of the metal phase of the catalysts were examined by temperature-programmed reduction (TPR). A sample containing 50 mg of each catalyst was inserted into a quartz cell and pretreated by nitrogen stream (99,9% purity), 75 mL min^–1^ at 300 °C for 3 h, to eliminate moisture and possible contaminants in the samples. After this step, the catalyst was subjected to the flow of a reducing mixture: 5% H_2_/95% N_2_ (75 mL min^–1^, 99.9% purity), with a heating rate of 10 °C. min^–1^, from room temperature until 1100 °C, maintaining this temperature for approximately 2 h. The equations used to calculate the degree of reduction of the peaks resulting from the programmed temperature reduction analysis are presented in the Supporting Information. The degree of dispersion and the metallic area of the catalysts were obtained by chemisorption in hydrogen, using the pulse titration technique, as well as the temperature reduction profiles were measured on the ChemBET Pulsar TPR/TPD equipment (Quantachrome Instruments, USA). With the catalyst reduced and the TCD (thermal conductivity detector) signal stabilized, a volume of 81 μL containing pure H_2_ is injected and chemisorbed. A variation in the TCD signal is observed after the signal stabilizes, indicating that all the H_2_ gas has been adsorbed and is only permeating through the sample. Thus, the amount of H_2_ adsorbed by the catalyst is calculated by selecting the peaks in which total or partial adsorption occurred. The amount of H_2_ adsorbed was used to calculate the metal surface area (*S*_m_ in m^2^ g^–1^ of metal) and the dispersion degree (*D* in % of metal), using the following equations

3 $$S_{m}=\frac{V_{H_{2}}\cdot FE\cdot N_{av}\cdot \partial_{m}}{V_{M}\cdot y_{Ni}\cdot m_{cat}}$$

where *V*_H2_ is the volume of chemisorbed H_2_ (μL), *V*_M_ is the molar volume under standard conditions (22.4 L mol^–1^), FE is the stoichiometric factor for dissociative chemisorption of hydrogen (2 H atoms/H_2_ molecule), *m*_cat_ is the catalyst mass (g), *N*_av_ is Avogadro’s constant (6.023 × 10^23^ H_2_ molecules/mol H_2_), *y*_Ni_ is the metal mass fraction in the catalyst, and ∂_m_ is the cross-sectional area of the metallic phase (0.0649 nm^2^/Ni atom). The degree of dispersion was calculated using the eq 4

4 $$D=\frac{N_{S}}{N_{T}}\times 100$$

where *N*_S_ (Ni atoms/g of catalyst) is the number of nickel atoms (active sites) on the surface and, therefore, accessible to H_2_ chemisorption, while *N*_T_ is the total number of atoms in the catalyst (Ni atoms/g of catalyst).

The carbon deposition on the nickel catalysts after reaction was evaluated by thermogravimetric analysis (TGA) coupled with differential thermal analysis (DTA), based on the mass loss profile. Approximately 4 mg of catalyst were heated from room temperature up to 800 °C under an oxidizing atmosphere (synthetic air, 21% O_2_/79% N_2_, 50 mL min^–1^) with 99.99% purity (White Martins), using a heating rate of 10 °C min^–1^. The analyses were performed in a Shimadzu TGA-50 instrument.

### 2.4 Catalytic Activity

The autothermal reforming of ethanol was carried out in a tubular quartz reactor placed inside a temperature-controlled furnace. For each run, 40 mg of catalyst were allocated in the reactor. The catalyst was previously reduced at 800 °C for 1 h 30 min under a flow of 50 mL min^–1^ of 5% H_2_/95% N_2_ (99.9% purity) stream. The ATR reaction was carried out under stoichiometric conditions, that is, with H_2_O/C_2_H_5_OH = 3 and O_2_/C_2_H_5_OH = 0.5 molar ratio at 600 °C. The mixture of C_2_H_5_OH and H_2_O was injected by a syringe pump (New Era Pump Systems, Inc.) at a flow rate of 27 μL min^–1^ and dragged by inert gas N_2_ at a flow rate of 720 mL min^–1^. The air flow rate was 14 mL min^–1^. Weight hourly space velocity (WHSV) was equal to 1300 h^–1^, calculated from eq 5. The products were analyzed in line using a gas chromatograph (PerkinElmer, Clarus 580) equipped with a flame ionization detector (FID) and thermal conductivity detector (TCD). Ethanol conversion (*X*_EtOH_) and product selectivity (*S*_i_) were calculated as described elsewhere.^35^ The reproducibility of the catalytic tests was evaluated in triplicate, under the same conditions previously described, using catalyst S2. The results obtained are presented in the Supporting Information. Because of the high uncertainties, water was not considered in product selectivity.

5 $$WHSV=\frac{{ṁ}_{total}}{W_{catalyst}}$$

Catalytic stability tests regarding coke formation were carried out under the following conditions: temperature equal to 600 °C, ratio H_2_O/C_2_H_5_OH = 3, ratio O_2_/C_2_H_5_OH = 0.5, and WHSV of 1300 h^–1^. Ethanol conversion and product distributions were monitored during the ethanol reforming reaction for approximately 500 min, using S1, S2, and S3 catalysts.

## 3 Results and Discussion

### 3.1 Field Emission Scanning Electron Microscope and Energy Dispersive Spectroscopy

Figure 2 presents the images obtained by FESEM of the S1, S2, S3, and S4 catalysts. A predominantly heterogeneous morphology, characterized by irregular agglomerates and variable porosity is observed, in agreement with the results of physical adsorption of N_2_ (Table 3). This distribution may be related to the synthesis process and the interaction between Ni and Al, suggesting the presence of particles with different sizes. Figure 1a,b, referring to catalysts S1 and S2, show morphological changes possibly induced by the heat treatment at 975 °C. This process led to the formation of larger agglomerates, resulting in a less homogeneous structure, with larger particles and a less uniform distribution of NiO on the surface. These characteristics may compromise the catalytic activity of the material. Analysis of the images corresponding to catalysts S3 and S4 (Figure 2c,d) reveals a nonuniform particle distribution or rather particle agglomerations.

**Figure 2:**
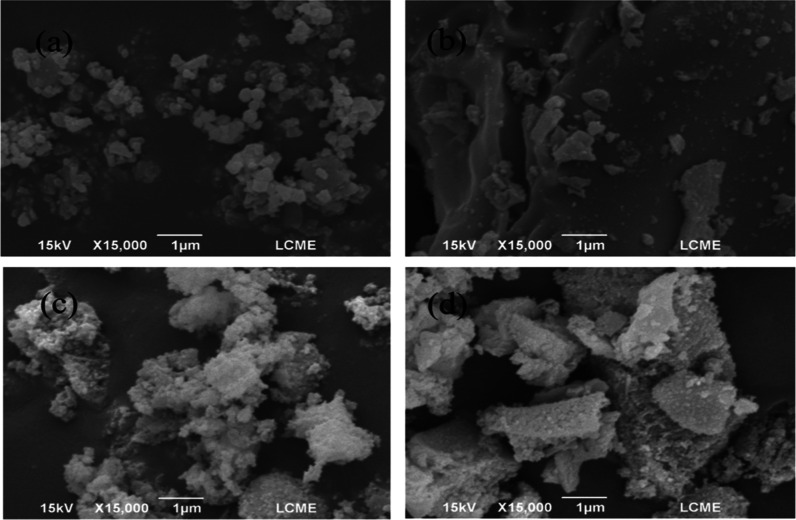
FESEM images for the samples: (a) S1, (b) S2, (c) S3 and (d) S4.

Figure 3 presents the spectra of the energy dispersive X-ray spectroscopy (EDX) analysis at the 250 μm scale. The results confirm the presence of the constituent elements of the catalysts: Al, Ni, O and Y. In particular, the peak associated with yttria overlaps with the peak corresponding to gold, as evidenced in the enlargement shown in Figure 4c,d. From the results obtained, it can be stated that the surface of the Al_2_O_3_ and Y_2_O_3_ support is covered by particles of the active phase.

**Figure 3:**
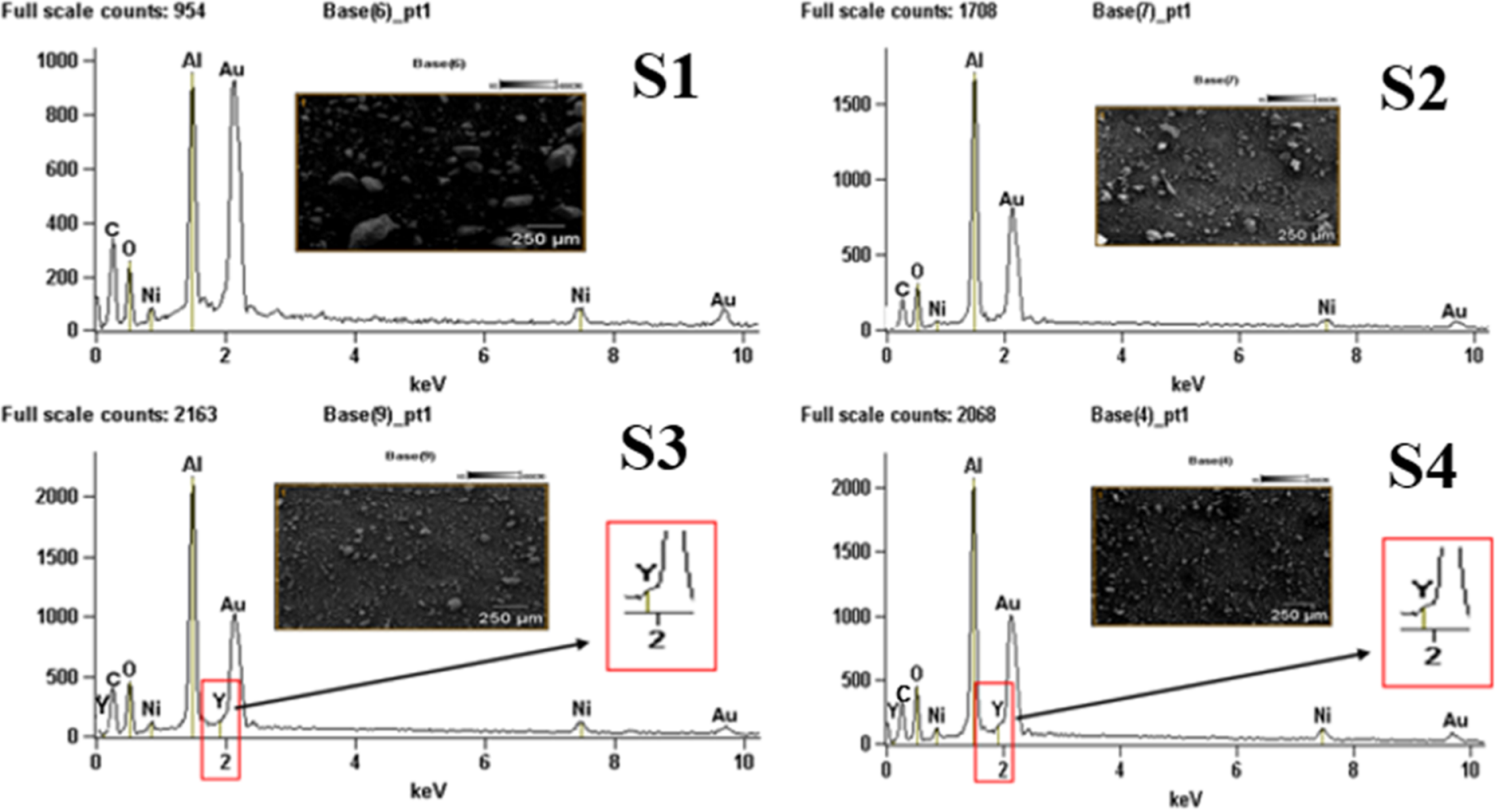
EDX spectra for samples: (a) S1, (b) S2, (c) S3 and (d) S4.

**Figure 4:**
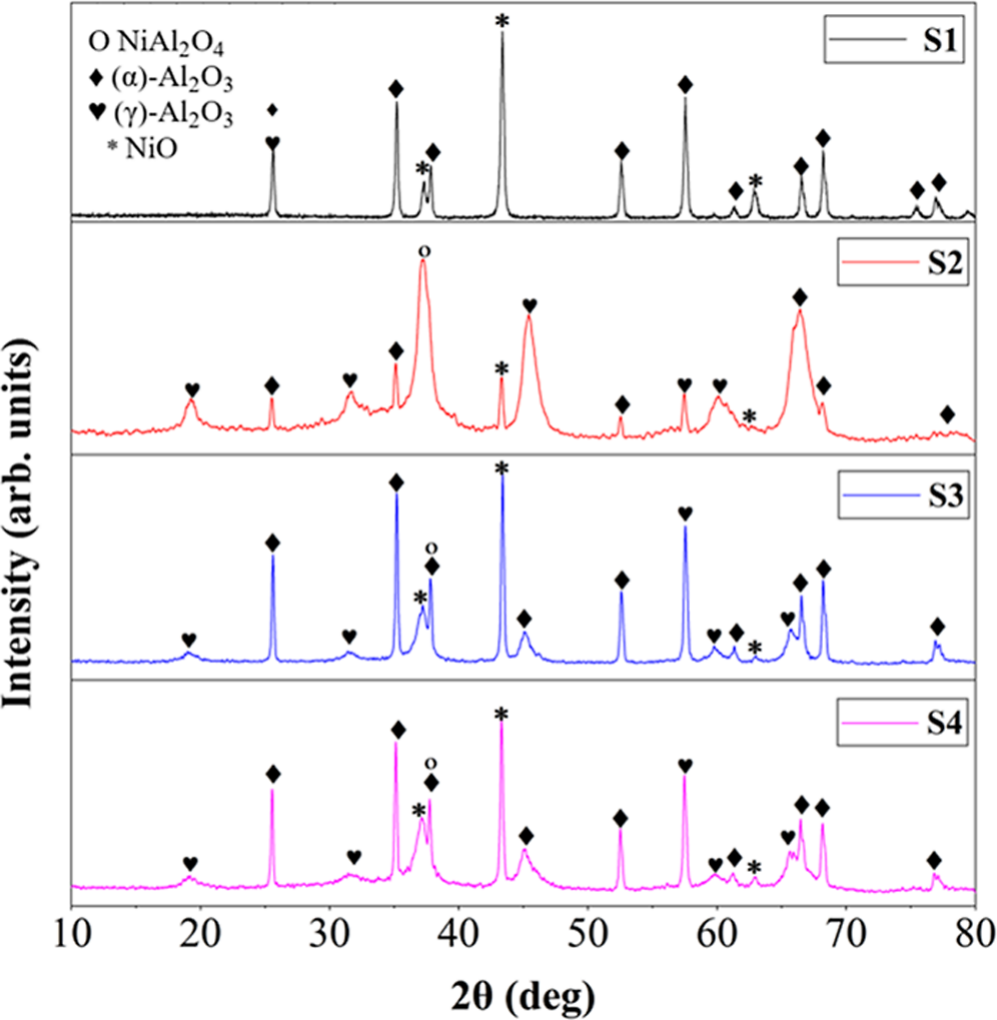
Diffractogram of catalysts S1, S2, S3 and S4.

### 3.2 X-ray Diffractometry

Figure 4 shows the results of the X-ray diffractometry technique of catalysts S1, S2, S3 and S4 after the calcination step. The spectra obtained reveal a polycrystalline structure, with the presence of the following crystalline phases: NiO (JCPDS 04-0835), NiAl_2_O_4_ (JCPDS 073-0239), γ-Al_2_O_3_ (JCPDS 01-079-1558) and α-Al_2_O_3_ (JCPDS 01-071-1123). All catalysts presented three characteristic peaks of NiO, located approximately at angles of 37.31°, 43.32° and 62.82°, with the exception of catalyst S2, which exhibited only two of these peaks. This difference may be related to the strong interaction between the active phase and the support, favoring the formation of NiAl_2_O_4_ spinel in sample S2, especially at high temperatures (975 °C), as indicated by the peak at 33.80° (eq 6). All catalysts showed evidence of NiAl_2_O_4_ spinel formation, except for catalyst S1, as will be discussed in the TPR analysis. It is worth noting that NiAl_2_O_4_ spinel usually has a blue coloration, as illustrated in Figure 5.^42^

6 $$NiO+{Al}_{2}O_{3}\to Ni{Al}_{2}O_{4}$$

**Figure 5:**
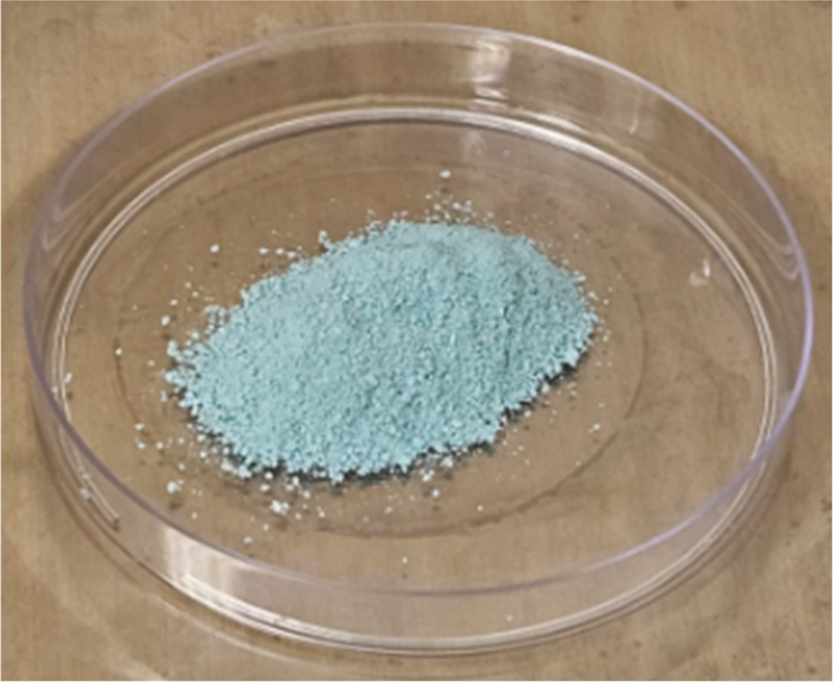
Image of catalyst S2, indicating the blue coloration typically found in nickel aluminate (NiAl_2_O_4_).

The average size of NiO crystallites (weighted by the areas attributed to this phase) is 35.32 nm for catalyst S2 (Table 2), which suggests a smaller dispersion of NiO particles. Although nickel spinel (NiAl_2_O_4_) was identified in all samples by the reduction profiles in the TPR result, the XRD analysis detected this phase only in sample S2, with a crystallite size of 33.80 nm, at an angle close to 37°, in agreement with the literature.^43^ In samples S3 and S4, which contain Y_2_O_3_, the size of the NiO crystallites is reduced (23.86 and 23.96 nm, respectively), in agreement with the literature.^10^ The addition of Y_2_O_3_ to the support reduces the interaction between NiO and the support in the form of NiAl_2_O_4_ spinel.^44^ Consequently, it was possible to identify more peaks related to NiO in catalysts S3 and S4, compared to catalyst S2, although all were supported on the same precursor (Al­(NO_3_)_3_·9H_2_O). It is observed that, in catalysts S3 and S4, the NiAl_2_O_4_ phase overlaps with the α-Al_2_O_3_ phase in the diffractometric peak located at 37.80°. These results corroborate those presented by Ranjbar (2019),^45^ indicating that the Al_2_O_3_ phase frequently overlaps with the NiAl_2_O_4_ phase at the 37.70° and 43.3° positions. According to Sun et al., and Yang et al.,^44,46^ this behavior may be related to the broadening of the peaks after the addition of Y_2_O_3_ and the overlapping of the Al_2_O_3_ and NiAl_2_O_4_ phases, which makes it difficult to distinguish between them. The absence of crystallinity peaks related to Y_2_O_3_ indicates its high dispersion in the synthesized support, corroborating the results on the textural properties of the catalysts (specific area and average pore size).^44^

**Table 2:** Average Crystallite Size of Synthesized Catalysts, Calculated Using the Scherrer Equation

average crystallite size (nm)				
sample	NiO	NiAl_2_O_3_	γ-Al_2_O_3_	α-Al_2_O_3_
S1	23			39
S2	35	34	9	47
S3	24	46	12	49
S4	24	44	20	47

In formulations S2, S3, and S4, several crystallinity peaks related to the γ-Al_2_O_3_ phase were identified, due to the heat treatment of the support in the calcination step. Myronyuk et al.,^47^ reported that the effects of calcination on the transformations of aluminum nitrate nonahydrate (Al­(NO_3_)_3_·9H_2_O) indicate that, in the range of 525–1000 °C, the alumina precursor forms the γ-Al_2_O_3_ phase, while the formation of the α-Al_2_O_3_ phase occurs at 1100 °C. Similar results were observed by Cava et al.,^48^ who identified the presence of the γ-Al_2_O_3_ and α-Al_2_O_3_ phases at distinct angles. These phases arose from the calcination of aluminum hydroxide (Al­(OH)_3_) at 1000 °C. Samples S2, S3, and S4 showed increasing γ-Al_2_O_3_ crystallite sizes (9, 12, and 20 nm, respectively). This suggests that the stabilization of γ-Al_2_O_3_ is favored by Y_2_O_3_. Furthermore, the growth of the γ-Al_2_O_3_ crystallite size in samples S3 and S4 may indicate a higher thermal resistance of this phase due to the structural modification caused by Y_2_O_3_.

### 3.3 Nitrogen Physisorption and Pulse Titration

Table 3 presents the results of the textural properties of the catalysts obtained from nitrogen physisorption the pulse titration technique. It determined the BET specific area, metallic area, degree of dispersion, and Ni^0^ crystallite size of the S1, S2, S3, and S4 catalysts. The influence of calcination temperature on the physicochemical properties was investigated by physical adsorption of N_2_ and chemisorption of H_2_. The incorporation of the active Ni phase into the support synthesized from the precursor Al­(NO_3_)_3_9H_2_O (converted to Al_2_O_3_ after calcination) resulted in a reduction in the specific surface area from 129 to 48 m^2^/g. Although X-ray diffraction analysis and scanning electron microscopy have not identified this behavior, previous studies have shown that this effect can be attributed to calcination at high temperatures, which promotes the contraction of the crystalline structure and the transformation of micropores into larger pores.^49^ Furthermore, the decrease in the area may be related to the partial blockage of the pores by the deposition of NiO particles on the surface of the support.^50^ The incorporation of Ni also increased the average pore diameter from 4 to 48 nm, comparing the Al_2_O_3_ support and the S2 catalyst. These changes can be influenced by the preparation steps, especially evaporation, drying, and calcination, which promote structural transformations and diffusion of chemical species in the support.^51^

**Table 3:** Properties of Catalysts Obtained by the Technique of Physical Adsorption of N_2_ (BET Method) and Chemisorption in H_2_ (Pulse Titration)

sample	average pore diameter (nm)	average pore volume (cm^3^/g)	BET specific area (m^2^ g^–1^)	metal area (m^2^ g^–1^ de catalyst)	degree of dispersion (%)	size of Ni^0^ crystallite (nm)
Al_2_O_3_	4	0.13	129			
(α)-Al_2_O_3_			7.50			
S1	57	0.10	7	0.17	0.26	130
S2	48	0.95	48	0.29	0.44	77
S3	28	0.11	17	0.15	0.23	150
S4	17	0.93	27	0.10	0.15	230

The S2 catalyst presented a larger metallic area and dispersion than the other synthesized catalysts. In the diffractogram of this material, it was possible to observe only two NiO peaks, which suggests a high metallic dispersion. This result is corroborated by the high specific area of the support, which favors such dispersion.^52^ Furthermore, based on the results obtained by the pulse titration technique, this catalyst presents a smaller Ni^0^ crystallite size (77 nm) after reduction, due to the strong interaction between NiO (NiO) and the alumina support (Al_2_O_3_), which leads to the formation of the spinel phase NiAl_2_O_3_.^53^ The addition of Y_2_O_3_ in catalysts S3 and S4 reduced the pore area and volume, possibly due to the deposition of this oxide on the inner surface of the support pores. This effect, although reducing the surface area, can inhibit the sintering of the metallic phase and increase the stability of the catalyst.^44^ The synthesized catalysts exhibited low metallic area and dispersion, associated with the larger size of the Ni^0^ crystals (Table 3). Since dispersion is inversely proportional to the particle size of the metallic phase, and this factor negatively influences the availability of active nickel.^52^ The addition of Y_2_O_3_ modifies the interaction between nickel and the support, increasing the strength of the Ni–Y_2_O_3_–Al_2_O_3_ interaction. This resulted in the formation of larger metallic particles and consequently affected the dispersion (Table 3). This effect occurs because nickel tends to sinter less, forming larger but more stable particles, which reduces the exposed metallic area.^44^ Although it reduces the metallic area and dispersion, this greater stability may contribute to the catalyst’s durability by limiting the availability of metallic nickel on the surface.^54^ In particular, the metallic area decreases due to the greater interaction of NiO with the support, where the reduction of NiO requires higher temperatures (Figure 5).^52^

### 3.4 Programmed Temperature reduction

The reduction profiles of the catalysts are shown in Figure 6. The S1 and S2, S3and S4 catalysts showed reduction peaks at temperatures of approximately 400, 500, 600, and 900 °C. For the S1 catalyst, hydrogen consumption in the reduction step occurred between 400 and 700 °C. In particular, three overlapping reduction peaks (curve 1, curve 2, and curve 3) can be observed at temperatures of 497, 587, and 639 °C. The different temperature peaks are associated with the reduction of NiO species to Ni^0^, with different degrees of interaction with the support. Therefore, the peak with the lowest temperature (curve 1) is associated with the reduction of NiO particles with less interaction with the support, while the peak with the highest temperature (curve 2 and curve 3) corresponds to the reduction of NiO particles that interact with the Al^3+^ species during the support impregnation step.^55^ The observed reduction profile is in agreement with other works published in the literature on the Ni/α-Al_2_O_3_ catalyst. Researchers such as Juan–Juan; Román-Martínez; Illán-Gómez; Pompeo et al.; Sanchez; Navarro; and Fierro,^56–58^ investigated the reduction profiles of the Ni/α-Al_2_O_3_ catalyst and observed a reduction in the 600 °C range attributed to the reduction of NiO species to Ni^0^, while the reduction peaks at high temperatures (>600 °C) were attributed to the reduction of nickel aluminate spinels (NiAl_2_O_4_).

**Figure 6:**
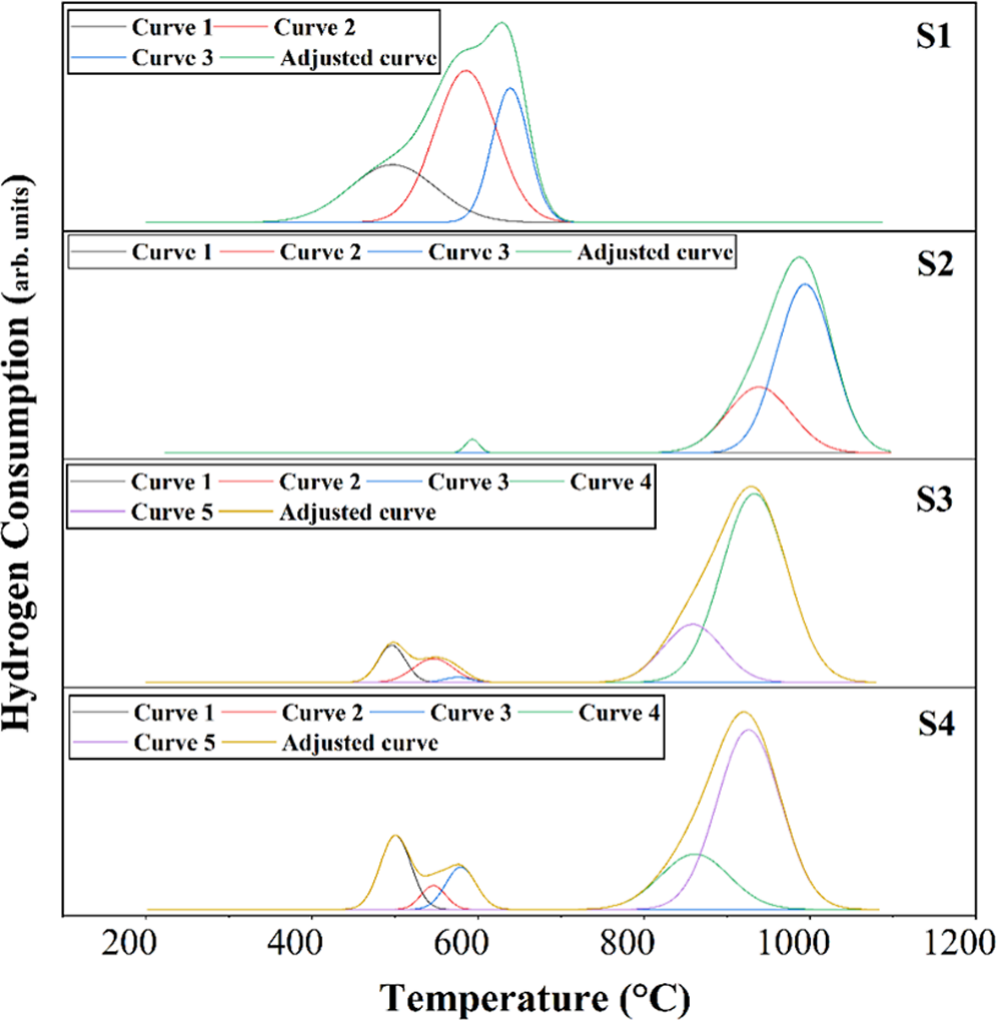
Reduction profiles of S1, S2, S3 and S4 catalysts.

Regarding the S2 catalyst, it is possible to observe that the peak associated with the reduction of the NiO species occurs with lower intensity (curve 1), corroborating previous results that indicate the presence of the α-Al_2_O_3_ phase in the support in a smaller proportion. In this catalyst, hydrogen consumption occurs mainly at high temperatures (800–1200 °C). In this temperature range, the presence of two overlapping peaks (curve 2 and curve 3) is observed, associated with the reduction of spinel NiAl_2_O_4_ (eq 7), as reported in several studies in the literature.^37,59,60^ The reduction temperature and peak width are indicative of the ease of reduction and the degree of interaction between different species, respectively. High reduction temperatures (above 600 °C) indicate that the reduction is difficult at the typical ethanol reforming temperature, while broad peaks indicate a high degree of interaction between the species and the support. The reduction peaks between 400 and 602 °C represent the reduction of NiO.^58,61^

7 $${NiAl}_{2}O_{4}+H_{2}\to {Ni}^{0}+{Al}_{2}O_{3}+H_{2}O$$

The S3 and S4 catalysts modified with yttria (Y_2_O_3_) have similar reduction profiles. The S2 formulation presents a smaller peak related to the NiO species. After the inclusion of yttria (Y_2_O_3_) as a promoter in the support, the appearance of two peaks (curve 1 and curve 2) is visible in the temperature range of 500–600 °C, attributed to the reduction of the NiO species. Therefore, the addition of yttria (Y_2_O_3_) in the support provided a shift of the TPR peaks at lower temperatures, accompanied by some broadening of the peaks, due to the lower rate of formation of NiAl_2_O_4_ spinel. In this sense, its addition promotes an increase in reducibility.^44^

Table 4 shows that all catalysts exhibit two reduction regions associated with NiO and the NiAl_2_O_4_ phase. In general, the spinel phase (NiAl_2_O_4_) predominates, with a relative contribution greater than 70%, indicating a strong interaction between nickel and the alumina support. Catalyst S2 shows almost all the reduction attributed to the NiAl_2_O_4_ phase (99%), revealing the strongest Ni–Al_2_O_3_ interaction, while S1, S3, and S4 exhibit lower proportions of this phase (74%, 88%, and 78%, respectively) and a greater presence of free NiO. The higher amounts of NiO in S3 and S4 suggests a higher fraction of more easily reducible species, which may favor the formation of active Ni^0^ at lower temperatures and potentially improve catalytic performance in reforming reactions.

**Table 4:** Degree of Reduction of NiO and Al_2_O_3_ Species

catalyst	peak temperature (°C)	species	area (arb. units)	degree of reduction (%)	relative contribution (%)
S1	497	NiO	853	26	26
	585	NiAl_2_O_4_	16	49	74
	639		847	25	
S2	483	NiO	13	1	1
	941	NiAl_2_O_4_	1952	99	99
S3	496	NiO	154	6	12
	546		144	5	
	575		21	1	
	933	NiAl_2_O_4_	1889	69	88
	858		521	19	
S4	501	NiO	279	12	22
	546		72	3	
	579		158	7	
	861	NiAl_2_O_4_	469	20	78
	926		1400	28	

### 3.5 Summary of Catalysts Physicochemical Properties

Table 5 presents the main physicochemical properties of the prepared catalysts. It can be observed that catalyst S1 presented the lowest specific surface area (7 m^2^ g^–1^). Catalyst S4 presented the largest crystallite size of metallic nickel (Ni^0^), 230 nm, resulting in the smallest metallic surface area (0.10 m^2^ g^–1^). Catalyst S3 exhibited an intermediate metallic surface area (0.15 m^2^ g^–1^) and a Ni^0^ crystallite size of 150 nm. Regarding NiO, catalyst S2 presented the largest crystallite size (35 nm) and also the highest specific surface area (48 m^2^ g^–1^), reflecting better dispersion of metallic particles (0.44%). It was also observed that, after the addition of the promoter, a shift occurred in the reduction peak associated with NiO at approximately 100 °C. This behavior can be verified by comparing unpromoted catalysts (range of 400–602 °C) with promoted catalysts (range of 500–600 °C), indicating greater metal–support interaction.

**Table 5:** Main Physicochemical Properties of Catalysts

sample	size of NiO crystallite (nm)	size of Ni^0^ crystallite (nm)	BET specific area (m^2^ g^–1^)	degree of dispersion (%)	metal area (m^2^ g^–1^ de catalyst)	NiO reduction temperature (°C)
S1	23	130	7	0.26	0.17	400–700
S2	35	77	48	0.44	0.29	400–602
S3	24	150	17	0.23	0.15	500–600
S4	24	230	27	0.15	0.10	500–600

### 3.6 Catalytic Activity

Table 6 presents a comparison of ethanol conversion during reactions carried out with catalysts S1, S2, S3, and S4, under a WHSV of 1300 h^–1^, measured after 10 min of reaction (initial steady state). All catalysts showed total conversion (100%) of ethanol. Although catalysts S1 and S2 promoted total conversion, the predominant reaction was ethanol dehydration, evidenced by the high selectivities for ethylene (C_2_H_4_), of 64.00% and 99.99%, respectively. According to Palma et al. and Rodríguez, Moreno, and Molina,^62,63^ ethylene is the main precursor of coke and is therefore responsible for catalytic deactivation. This reaction is favored by the presence of acidic sites on the Al_2_O_3_ support. In the case of catalyst S1, a selectivity of 15% for H_2_ and 21% for CO_2_ was observed, products resulting from the ethanol reforming reaction. Although not the predominant reaction, its occurrence is related to the acidic sites of the support and the high proportion of hydrogen atoms in the ethylene molecule, which limits the significant formation of H_2_ in the process.^64^

**Table 6:** Ethanol Conversion and Selectivity of the Reactions Products with Catalysts S1, S2, S3 and S4 in WHSV of 1300 h^–1^

		product selectivity (S) (%)					
catalyst	conversion of ethanol (%)	H_2_	CO	CO_2_	C_2_H_4_O	C_3_H_6_O	C_2_H_4_
S1	100.00	15	0.00	21.00	0.00	0.00	64.00
S2	100.00	0.00	0.00	0.10	0.00	0.00	99.99
S3	100.00	40.00	1.00	59.00	0.00	0.00	0.00
S4	100.00	1.00	0.00	11.00	0.00	0.00	88.00

When comparing the catalysts doped with Y_2_O_3_, catalyst S3 showed better catalytic performance than S4, with selectivities of 40% for H_2_ and 59% for CO_2_. These results indicate that S3 favors the ethanol reforming reaction, showing greater catalytic activity associated with a more adequate distribution of metal sites.^65–67^ On the other hand, increasing the Y_2_O_3_ load to 2% in catalyst S4 resulted in the formation of larger metallic particles, approximately 230 nm in diameter (Table 3), due to a reduction in nickel dispersion. This effect occurs because Y_2_O_3_ tends to partially coat the support surface, blocking pores and restricting access to the metal’s active sites. Consequently, there is a limitation in the cleavage of the C–C bond of the ethanol molecule, favoring the dehydration reaction, forming ethylene,^33,52^ as evidenced in Table 6.

### 3.7 Trade-Off Analysis

The results demonstrated that dispersion limits the addition of Y_2_O_3_. Incorporation of levels higher than 1% reduces dispersion and metallic area, which negatively affects the catalytic activity. In the case of catalyst S4, containing 2% Y_2_O_3_, a greater favoring of ethanol dehydration reactions was observed, resulting predominantly in the formation of ethylene, a compound recognized as a coke precursor. It is likely that the excess Y_2_O_3_ (2%) caused partial blockage of the pores of the Al_2_O_3_ support, leading to agglomeration of the active phase and, consequently, a decrease in dispersion and metallic area.

Therefore, catalyst S3 showed superior performance in ethanol conversion and hydrogen selectivity. In contrast, catalyst S4, which has an addition of yttria at a level greater than 1%, showed a reduction in the metallic area and dispersion of the active phase, as well as the occurrence of undesirable ethanol dehydration reaction. For this reason, only catalysts S1, S2, and S3 were selected for long-term stability tests regarding coke formation, as will be discussed in the next section.

### 3.8 Catalytic Stability

Figure 7 shows the ethanol conversion and the selectivities of the products (S) obtained with catalyst S1. The conversion remained at 100% over time. The main products formed were C_2_H_4_, H_2_, and CO_2_. It was observed that the selectivity for H_2_ and CO_2_ decreased with reaction time, while the selectivity for ethylene (C_2_H_4_) increased, reaching 95%, indicating the predominance of the ethanol dehydration pathway, which was the predominant reaction in the system.

**Figure 7:**
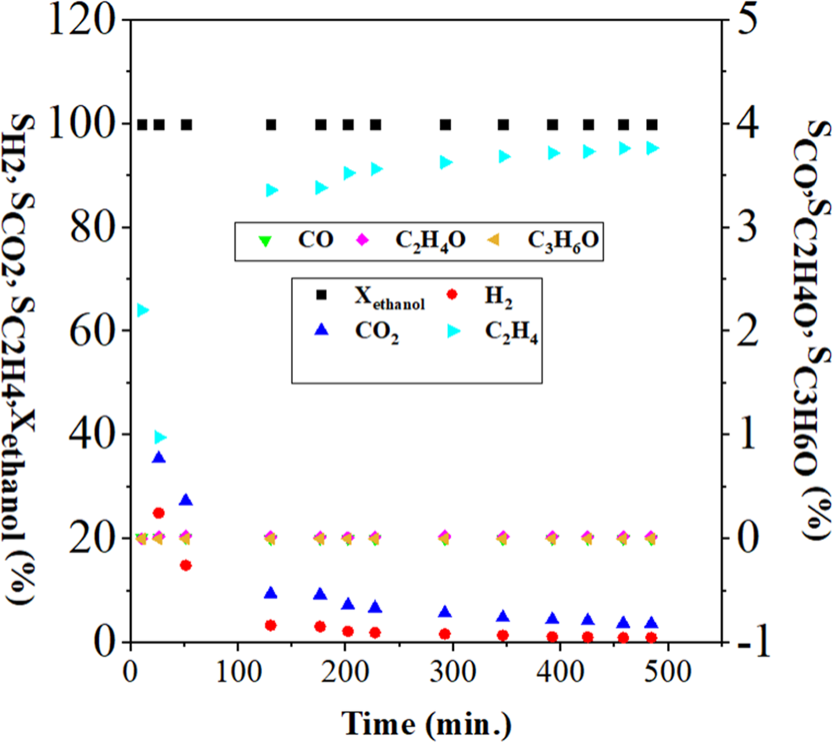
Ethanol conversion and selectivity of reaction products with the S1 catalyst.

In Figure 8, it can be observed that the conversion of ethanol with catalyst S2 was 100%. The absence of H_2_ and CO_2_ among the products indicates that neither ethanol reforming nor gas–water shift reactions occurred. The selectivity for ethylene was 99.99%, highlighting the influence of dehydration reactions on catalyst deactivation. Ethylene decomposition was identified as the main route for coke formation.^35,68^

**Figure 8:**
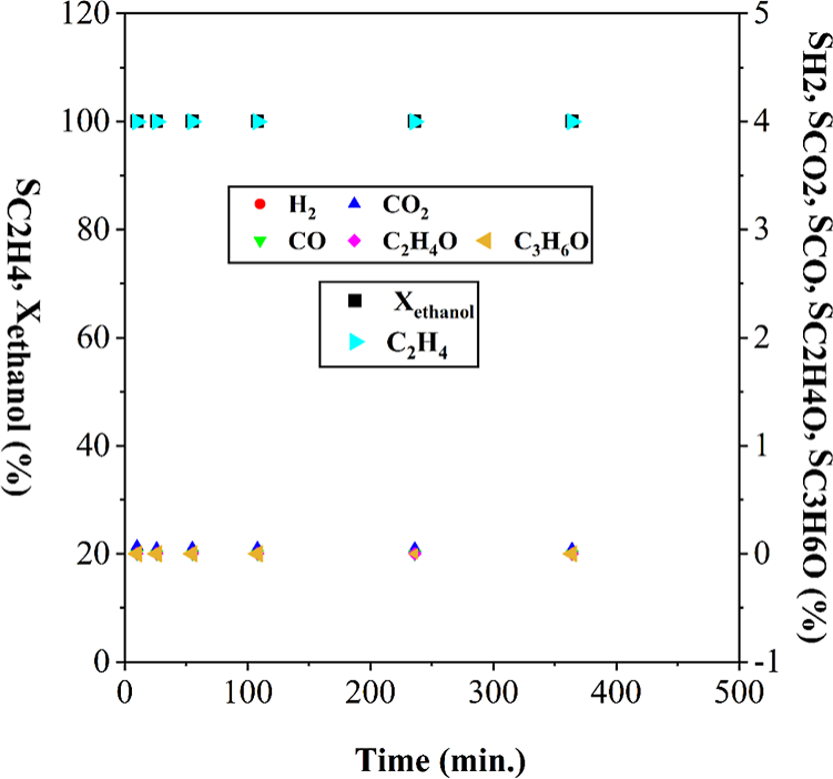
Ethanol conversion and selectivity of reaction products with the S2 catalyst.

Figure 9 shows that catalyst S3 maintained total ethanol conversion, exhibiting the highest H_2_ and CO_2_ production rates, attributed to the presence of Ni^0^ metallic species on the surface. The addition of Y_2_O_3_ favored the reduction of NiO to Ni^0^, reducing the formation of undesirable byproducts such as ethylene. In the first 100 min of the reaction, the selectivity for ethylene was less than 1%, indicating the predominance of ethanol reforming and gas–water shift reactions, as evidenced by the high concentrations of H_2_ and CO_2_. However, after 400 min, the selectivity for ethylene increased, reaching approximately 77%.^44^ The neutralization of acidic sites by Y_2_O_3_ inhibited secondary reactions, making the catalyst more efficient in reforming ethanol in the initial stages of the reaction.^25^ Furthermore, the literature reports that the formation of a NiAl_2_O_4_ spinel phase during catalyst preparation is beneficial for promoting high dispersion of Ni in the reduced phase, which, in turn, improves conversion and maximizes hydrogen production.^69,70^

**Figure 9:**
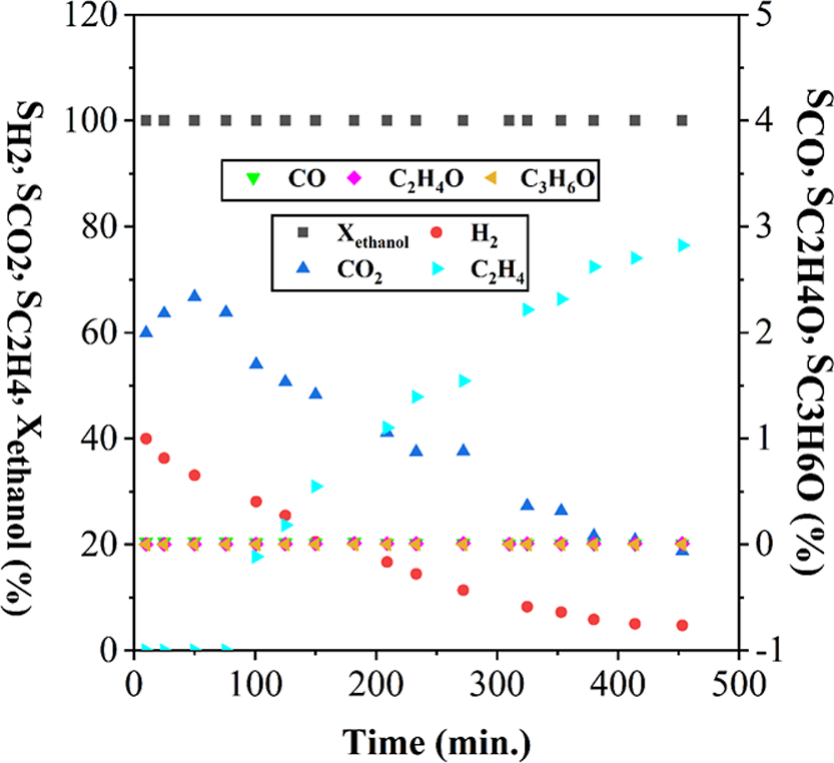
Ethanol conversion and selectivity of reaction products with the S3 catalyst.

### 3.9 Characterization of Catalysts after Reactions

The increase of ethylene selectivity over time results in coke formation on the catalyst surface, which can be confirmed by FEG-SEM images of the surfaces after the reaction. These images show the formation of filamentous carbon in samples S1, S2, and S3 (Figure 10), a result that corroborates the findings of Srisiriwat et al.^16^ In particular, these authors observed significant filamentous carbon deposits in unpromoted NiAl catalysts; in contrast, the promoted catalysts (NiCeAl, NiZrAl, and NiCeZrAl) exhibited excellent resistance to carbon formation, with only small amounts of filamentous carbon observed in the TEM images. These findings are aligned with the results of this work. To evaluate the effect of the Y_2_O_3_ promoter on the carbon formation of the Ni/Al_2_O_3_ catalysts, the thermogravimetric profiles of the recorded events were analyzed (Figure 11).

**Figure 10:**
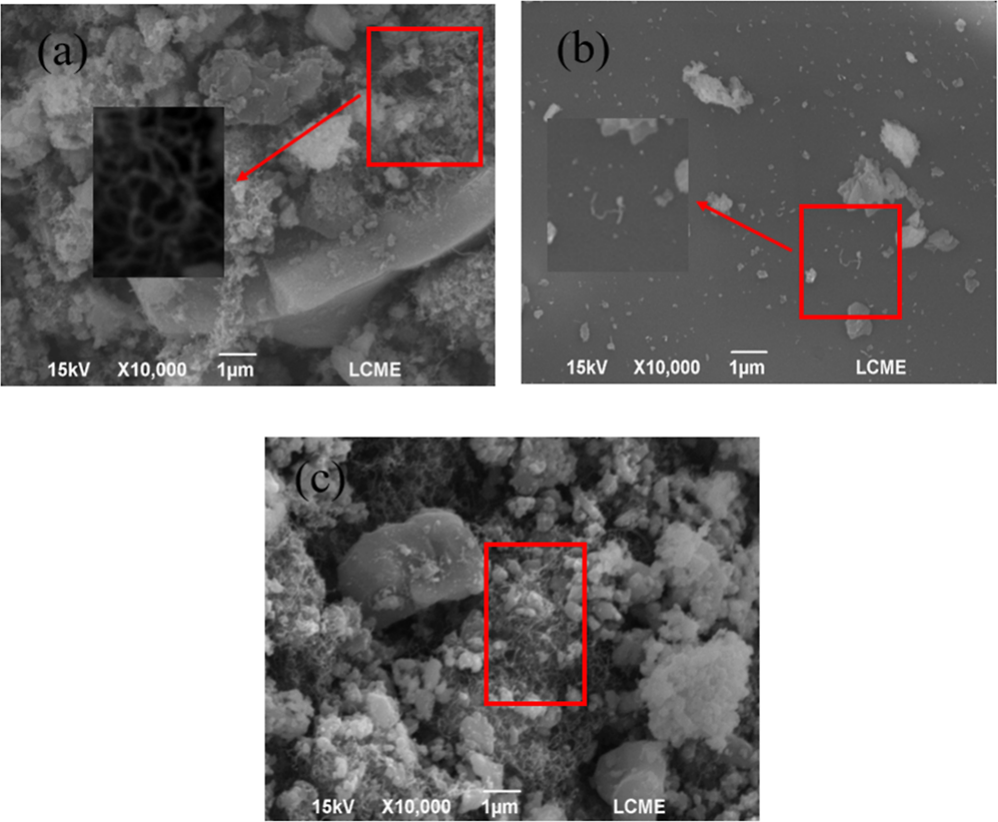
FESEM images after the catalyst reaction, (a) S1, (b) S2 and (c) S3.

**Figure 11:**
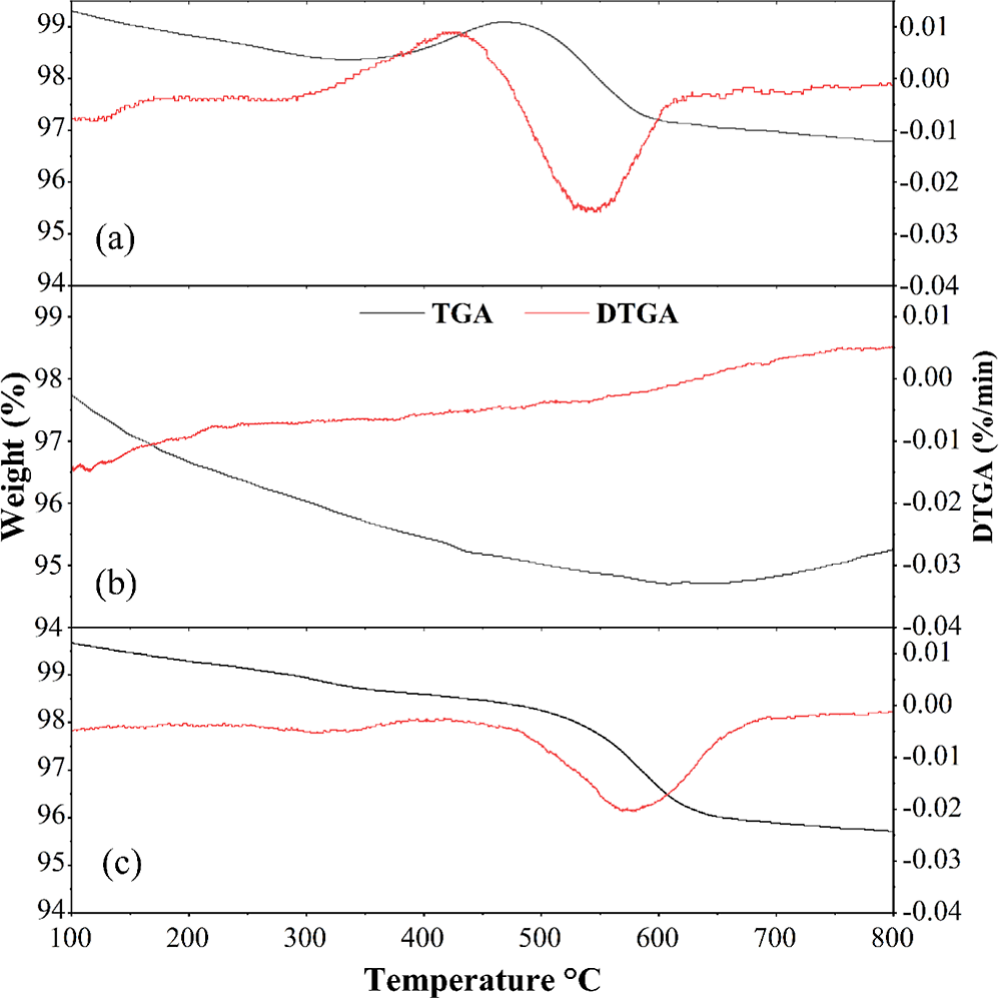
TG and DTG profiles of the catalysts tested after the reforming reactions: (a) S1, (b) S2, and (c) S3.

The mass gain observed at a temperature of 350 °C (Figure 11a) and at 800 °C (Figure 11b) is possibly associated with the oxidation of Ni^0^ to NiO in the presence of air.^71–73^ On the other hand, the material doped with Y_2_O_3_ (Figure 11c) exhibits a mass loss of 550 °C, attributed to carbon combustion. In general, mass losses occurring at temperatures below approximately 550 °C are related to the oxidation of less structured carbon species, such as unencapsulated filamentous carbon or monatomic carbon present on the catalyst surface. Mass losses occurring above 550 °C are attributed to the oxidation of more resistant forms, typically graphitic carbon.^74,75^ The S3 catalyst demonstrated greater thermal stability compared to the others, as its metallic particles did not undergo oxidation. Y_2_O_3_ contributes to this stability due to its high oxygen mobility, interaction with Ni, and maintenance of the reducibility of the metallic particles.^62^ All catalysts formed approximately 1 mg_coke_/mg_catalyst_, with the reaction conditions being decisive for the low deposition. Studies indicate that temperatures below 650 °C and H_2_O/C_2_H_5_OH molar ratios ≥ 2 can minimize coke formation.^32,70^ Furthermore, resistance to carbon deactivation may be related to the formation of spinel NiAl_2_O_4_ at high calcination temperatures.^27,29^

## 4 Conclusions

In this work, the production of nickel catalysts supported on alumina with the addition of Y_2_O_3_ to the support (Ni/Y_2_O_3_–Al_2_O_3_), prepared by the wet impregnation method, with high-temperature calcination (975 °C), was studied for the autothermal reforming of ethanol. The main focus of the study was to evaluate the effect of Y_2_O_3_ addition on catalyst stability, particularly regarding its resistance to deactivation by coke formation during the autothermal reforming of ethanol, as well as the influence of the NiAl_2_O_4_ spinel on the catalyst structure.

The results demonstrate that the composition and modification of the support play a crucial role in the structural, textural, and catalytic properties of the materials studied. The characterization results showed the formation of NiO, NiAl_2_O_4_, and γ-Al_2_O_3_ phases, with the NiAl_2_O_4_ spinel phase being detected mainly in the S2 catalyst, indicating strong interaction between nickel and alumina, and its reduction occurring at higher temperatures. The addition of Y_2_O_3_ (S3 and S4) reduced the NiO crystallite size and increased its reducibility.

In catalytic tests, catalyst S1 showed complete ethanol conversion but underwent deactivation due to coke formation originating from ethylene. Catalyst S2 did not exhibit hydrogen selectivity due to the predominance of dehydration reactions. In contrast, catalyst S3, containing Y_2_O_3_, demonstrated the best performance, with complete ethanol conversion, high H_2_ production, and reduced formation of undesirable byproducts in the first 100 min of reaction. These results confirm the promoting effect of Y_2_O_3_, which acts in neutralizing acidic sites and stabilizing the active metal phase. Therefore, it can be concluded that incorporating Y_2_O_3_ into the Al_2_O_3_ support in Ni catalysts not only improves the reducibility and dispersion of nickel, but also increases thermal stability and resistance to coke formation throughout the process, resulting in a more efficient material for the autothermal reforming of ethanol aimed at hydrogen production.

## Supplementary Material



## Notes

Additional experimental details, including Rietveld refinement and reproducibility of reaction results (PDF)
