# Supplementary material for: Effect of the Y2O3 Promoter on the Structure of the Ni/Al2O3 Catalyst for Ethanol Autothermal Reforming
Source: ACS Omega. 2025 Dec 11;10(50):61972–83. doi: 10.1021/acsomega.5c08809 (PMC12750206; doi:10.1021/acsomega.5c08809)
Supplement: Supplementary file 1 [file ao5c08809_si_001.pdf]

Effect of the Y_2O_3 promoter on the structure of the Ni/Al_2O_3 catalyst for ethanol autothermal reforming

Augusto P. Cambunda [1], Maíra O. Palm [2], Diego A. Duarte [2,3], Rafael C. Catapan [2,4], and Bruno F. Oechsler [1, *]

[1]Graduate Program in Chemical Engineering (POSENQ), Federal University of Santa Catarina, Florianópolis, 88040-900, Santa Catarina, Brazil.

[2]Graduate Program in Mechanical Science and Engineering (POSECM), Federal University of Santa Catarina, 89219-600, Joinville, Santa Catarina, Brazil

[3]Physics Graduate Program (PPGF), Santa Catarina State University of

, 89219-710, Joinville, Santa Catarina, Brazil.

[4]Graduate Program in Mechanical Engineering (POSMEC), Federal University of Santa Catarina, 88040-900, Florianópolis, Santa Catarina, Brazil

[*] Corresponding author: b.oechsler@ufsc.br

SUPPLEMENTARY MATERIAL

Rietveld refinement

Table S1 presents the results of the Rietveld refinement performed using the X'Pert HighScore Plus software, in which the R_{wp} values obtained for the NiO (JCPDS 04-0835) and $NiAl_2O_4$ (JCPDS 073-0239) phases were greater than 40%, a value mainly associated with the presence of multiple phases in the catalysts and the occurrence of amorphous regions that hinder the adjustment of the crystallographic model. According to Silva (2022)[1] and Nishihora[2], the Rietveld method is highly sensitive to amorphous phases, non-crystalline regions, and intense peak overlap, factors that can significantly increase the R_{wp} even when the structural model is adequate. Nevertheless, the refined model is chemically consistent, showing coherence with the temperature-programmed reduction (TPR) results, which corroborate the presence and reductive behavior of the NiO and $NiAl_2O_3$ phases (Figure 5).

Table S1 - Adjustment determined by the Rietveld refinement method of the X-ray diffractograms of the catalysts.

	**S1**	**S2**		**S3**		**S4**	
**Goodness of fit (G)**	NiO	NiO	$NiAl_2O_4$	NiO	$NiAl_2O_4$	NiO	$NiAl_2O_4$
$\boldsymbol{R_{wp}}$ **(%)**	60	49	42	59	57	50	48
$\boldsymbol{R_{exp}}$ **(%)**	15	13	13	13	13	13	13
$\boldsymbol{x^2}$	16	14	10	21	19	15	14

Where: $R_{wp} =$ weighted profile R-factor, $R_{exp} =$ expected profile R-factor x^2 is the chi-square value, referred to as the goodness of fit (G), and is given by the following equation:

$$x^2 = \frac{R_{wp}}{R_{exp}} \quad (1)$$

Degree of reduction

According to Schmal (2011)[3], the degree of reduction of catalysts (α) can be calculated from the following equation:

$$\alpha = \frac{m_{H_2}}{m_{H_2}^{\text{Theoretical}}} \times 100 \tag{2}$$

where $m_{H_2}$is the mass of hydrogen consumed during the reduction of nickel-containing species (represented as NiX, where X can be nickel oxide or nickel aluminate), while $m_{H_2}^{Theoretical}$ represents the theoretical mass of hydrogen consumed in the reduction, assuming that the nickel added to the support by impregnation is in the form of nickel oxide (NiO). The area of a peak during the reduction test is directly proportional to the mass of hydrogen consumed for the reduction of the respective nickel species and, consequently, is proportional to the mass of metallic nickel produced. Therefore, the equation can be rewritten as:

$$\alpha = \frac{m_{Ni}}{m_{Ni}^{\text{Theoretical}}} \times 100 \tag{3}$$

The peak areas calculated from the deconvolution of the reduction profiles can be used to calculate the mass fractions (y_{Ni}^{NiX}) relative to metallic nickel obtained through the reduction of the respective nickel species (NiO or $NiAl_2O_4$), that is:

$$y_{Ni}^{NiX} = \frac{A_{NiX}}{\mathrm{A}_{\text{total}}} \times 100 \tag{4}$$

where A_{NiX} is the area corresponding to the reduction peak of the NiX species (NiO or $NiAl_2O_4$) and A_{total} is the sum of the areas of all peaks present in the reduction profile. With the respective fractions and the theoretical mass of nickel in the catalyst, it is possible to estimate the degree of reduction of the nickel species as the mass fraction (y_{Ni}^{NiX}) mentioned above, since:

$$m_{Ni} = y_{Ni}^{NiX} m_{Ni}^{\text{Theoretical}} \quad (5)$$

Reproducibility of catalytic tests

The mean and standard deviation of ethanol conversion and the selectivity of the products formed during the reaction with catalyst S2 were presented in Table S2.

Table S2 – Mean and standard deviation of reaction results obtained with catalyst S2

	Measure 1	Measure 2	Measure 3	Average	Standard deviation
$X_{ethanol}$	99.99	99.99	99.99	99.99	0.00
SH2	0.00	0.00	0.00	0.00	0.00
S_{CO2}	0.00	9.94	0.00	3.31	5,70
S_{CO}	0.00	0.00	0.00	0.00	0.00
S_{C2H4O}	0.00	0.00	0.00	0,00	0.00
S_{C3H6O}	0.00	0.00	0.00	0.00	0.00
S_{C2H4}	99.91	89.97	99.93	96.60	5.74

Reference

(1) Araldi da Silva, B.; da Silva, J. C. G.; Gómez González, S. Y.; Moreira, R. de F. P. M.; Peralta, R. A.; Hotza, D.; De Noni Junior, A. Synergetic One-Step Synthesis of SiC/SiOC/TiO2 Composites for Visible-Light-Driven Hydrogen Generation from Methanol Reforming. *Ceram Int* **2022**, *48* (22), 32917–32928. https://doi.org/10.1016/j.ceramint.2022.07.221.

(2) Nishihora, R. K.; Rudolph, E.; Quadri, M. G. N.; Hotza, D.; Rezwan, K.; Wilhelm, M. Asymmetric Mullite Membranes Manufactured by Phase-Inversion Tape Casting from Polymethylsiloxane and Aluminum Diacetate. *J Memb Sci* **2019**, *581*, 421–429. https://doi.org/10.1016/j.memsci.2019.03.047.

(3) SCHMAL, M. *Catálise Heterogênea*, 1ª.; Synergia, Ed.; Rio de Janeiro, 2011.
